# Down-Regulation of Gab1 Inhibits Cell Proliferation and Migration in Hilar Cholangiocarcinoma

**DOI:** 10.1371/journal.pone.0081347

**Published:** 2013-11-28

**Authors:** Haiquan Sang, Tingting Li, Hangyu Li, Jingang Liu

**Affiliations:** 1 Department of General Surgery, The Fourth Affiliated Hospital of China Medical University, Shenyang, Liaoning, PR China; 2 Department of Clinical Genetics, Shengjing Hospital, China Medical University, Shenyang, Liaoning, PR China; 3 Aab Cardiovascular Research Institute and Department of Medicine, University of Rochester School of Medicine and Dentistry, Rochester, New York, United States of America; 4 Department of General Surgery, Shengjing Hospital, China Medical University, Shenyang, Liaoning, PR China; Emory University, United States of America

## Abstract

Hilar cholangiocarcinoma is a highly aggressive malignancy originating from the hilar biliary duct epithelium. Due to few effective comprehensive treatments, the prognosis of hilar cholangiocarcinoma is poor. In this study, immunohistochemistry was first used to detect and analyze the expression of Gab1, VEGFR-2, and MMP-9 in hilar cholangiocarcinoma solid tumors and the relationships to the clinical pathological features. Furthermore, Gab1 and VEGFR-2 siRNA were used to interfere the hilar cholangiocarcinoma cell line ICBD-1 and then detect the PI3K/Akt signaling pathway, MMP-9 levels and malignant biological behaviors of tumor cells. The data showed that 1. Gab1, VEGFR-2, and MMP-9 were highly expressed and positively correlated with each other in hilar cholangiocarcinoma tissues, which were related to lymph node metastasis and differentiation. 2. After Gab1 or VEGFR-2 siRNA interference, PI3K/Akt pathway activity and MMP-9 levels were decreased in ICBD-1 cells. At the same time, cell proliferation decreased, cell cycle arrested in G1 phase, apoptosis increased and invasion decreased. These results suggest that the expression of Gab1, VEGFR-2, and MMP-9 are significantly related to the malignant biological behavior of hilar cholangiocarcinoma. Gab1 regulates growth, apoptosis and invasion through the VEGFR-2/Gab1/PI3K/Akt signaling pathway in hilar cholangiocarcinoma cells and influences the invasion of tumor cells via MMP-9.

## Introduction

Hilar cholangiocarcinoma originates from the hilar biliary duct epithelium and is highly invasive, accounting for 50%-60% of cholangiocarcinomas [1,2]. Hilar cholangiocarcinoma is the most common malignant tumor of the biliary system [2]. Epidemiologic study results have shown an increasing incidence of hilar cholangiocarcinoma, which has become one of the major malignancies threatening human health in recent years [3]. Radical surgical resection is the most effective therapy available to treat hilar cholangiocarcinoma [4]. However, hilar cholangiocarcinoma is difficult to detect in the early stage due to its unique anatomical position and pathological characteristics. Most clinical patients have middle or advanced stage hilar cholangiocarcinoma with metastatic invasion to adjacent tissues by the time they seek medical care, resulting in a low radical resection rate [5,6]. As a result of frequently observed local recurrence, the postoperative 5-year survival rate is <30%, even in patients who undergo radical surgery [4,7]. The survival rate cannot be prolonged by radiotherapy or chemotherapy [8,9]. For inoperable patients, the median survival is 6-12 months [10] and the overall 5-year survival rate is <5% [11]. Effective comprehensive therapies have not yet been established to treat hilar cholangiocarcinoma. To improve the therapeutic effects of hilar cholangiocarcinoma, mechanisms regarding molecular regulation associated with occurrence, infiltration and metastasis should be studied in addition to early intervention.

The Gab protein family is a type of adapter protein (also known as scaffolding or docking proteins) which is capable of coupling with growth factor receptor binding protein 2 (Grb2). Gab1 is the most common and is widely distributed in mammals [12]. It is located in the cytoplasm and recruited to the cell membrane after being activated to bind with several activated signal receptors, resulting in its own activation [13]. Gab1, which is widely distributed in various body tissues, is capable of promoting cell growth, differentiation and migration and is involved in control of cell apoptosis [14]. Studies have found that Gab1 up-regulates proto-oncogene expression via participation in amplification of signaling pathways related to tumor biological behaviors and plays a role in the occurrence and development of tumors [15,16]. However, there have been no reports describing the relationship between Gab1 and biological behaviors of hilar cholangiocarcinoma to date.

Growth, invasion and metastasis of malignant tumors depend on neonatal blood vessels and lymphatic vessels in which VEGFR-2 plays a key role [17,18]. Studies have shown that VEGFR-2 can contribute to the occurrence, invasion and metastasis of tumors [19,20]. Damage to the extracellular matrix (ECM) and basement membrane is required for malignancy invasion and metastasis. MMP-9 is capable of degrading ECM and the basement membrane and promoting tumor invasion and metastasis, playing a key role in the above damage processes [21,22]. As reported in the literature, the adapter protein Gab1 can mediate VEGFR-2 to activate PI3K/Akt and advance cell invasion [23], while MMP-9 is an important downstream target protein of PI3K/Akt [24]. We presumed that Gab1 might affect the biological behaviors of tumor cells by mediating VEGFR-2 via PI3K/Akt and could influence the invasion and metastasis of tumor cells via MMP-9.

In this study, we detected VEGFR-2, Gab1 and MMP-9 expression in solid tumor hilar cholangiocarcinoma tissues to study their interactions and relationships with the clinical and pathological characteristics of hilar cholangiocarcinoma. We further investigated the signaling pathways that Gab1 was involved in and the effects of Gab1 on the biological behaviors of hilar cholangiocarcinoma using ICBD-1 cells.

## Materials and Methods

### Subjects

A total of 49 paraffin block specimens were selected from patients who underwent surgical resection at Shengjing Hospital of China Medical University from 2010 to 2011. Cases were pathologically confirmed as having hilar cholangiocarcinoma, and complete clinical and pathological data were provided. All patients did not receive chemotherapy or radiotherapy prior to surgery and were pathologically confirmed as having adenocarcinoma after surgery. A total of 20 paraffin block specimens of the biliary duct resected during partial hepatectomy from patients with intrahepatic bile duct stones pathologically confirmed as having chronic inflammation who were admitted during the same period were used as controls. Medical scientific research and new technology ethics committee of Shengjing Hospital of China Medical University approved this study. All participants provided their written consent before this study, and all clinical investigation have been conducted according to the principles expressed in the Declaration of Helsinki.

### Cells and Reagents

Human hilar cholangiocarcinoma ICBD-1 cells were purchased from Shanghai Aiyan Biotech Co., Ltd. Tumor cells were cultured in DMEM containing 10% fetal bovine serum at 37°C, 5% CO2, and saturated humidity with one passage every 3d. Cells in the logarithmic growth phase were used for experiments. Reagents were obtained as follows: Gab1 and VEGFR-2 monoclonal antibodies from Cell Signaling Technology; MMP-9 polyclonal antibody from ProteinTech; SABC Immunohistochemical Staining Kit and DAB Coloring Kit from Wuhan Boster Bio-engineering Co., Ltd.; DMEM (12100-46) from Gibco; TIAN Script RT Kit and RNA simple Total RNA Kit from Tiangen Biotech (Beijing) Co., Ltd.; Super M-MLV (PR6502) reverse transcriptase from BioTeke; Liposome 2000 (1024993) from Invitrogen; Cell Cycle Test Kit (C1052) from Beyotime; Cell Apoptosis Test Kit (KGA106) from KeyGEN Biotech; and MTT and DMSO from Sigma. Primers were synthesized by Sangon Biotech (Shanghai) Co., Ltd.

### Immunohistochemistry

The SABC immunohistochemical assay was used. All experimental procedures were performed according to specifications in the product instructions. Known positive breast cancer sections served as positive controls. Negative controls were added using PBS instead of primary antibody. Images were evaluated independently by two pathologists. Cells with brown particles were assessed as positive cells; stained cells with brown particles in the cytoplasm and on the cell membrane were assessed as Gab1-positive or VEGFR-2-positive cells, while those with brown particles in the cytoplasm were assessed as MMP-9-positive cells. Nikon Ti high-resolution color pathology imaging and character report management system was used for mean optical density (OD) quantification of Gab1, VEGFR-2 and MMP-9 expression. Each section was observed in 5 random, complete, non-overlapping, high-power fields (×400). The mean OD of positive reactions in each field was measured, and values from 5 fields were averaged to provide a final measurement result for each section.

### Cell Culture and Expression Detection

ICBD-1 cells were cultured in DMEM containing 10% fetal bovine serum at 37°C, 5% CO2, and a saturated humidity. Total RNA (using a RNA simple Total RNA Kit) and protein were extracted according to the experimental design, and the RNA and protein expression of Gab1 and VEGFR-2 were detected by RT-PCR (TIAN Script RT Kit) and Western Blot. RT-PCR primers were as follows: Gab1-F: ATCAGAAACGCCAGCGAAGA, Gab1-R: TCAGATACCACAAAGCACCA, VEGFR-2-F: CCAATAATCAGAGTGGCAGTG, and VEGFR-2-R: ATAGACATAAATGACCGAGGC.

### siRNA Interference

#### siRNA Design and Transfection

Four siRNA interference sequences of Gab1 or VEGFR-2, 1 irrelevant interference sequence (negative control) and 1 GAPDH interference sequence (positive control) were designed separately based on GenBank. Interference sequences were synthesized by Sangon Biotech (Shanghai) Co., Ltd. Cells were transfected with siRNA using Lipofectamine 2000, and RNA and protein were extracted from cells after a 48 h transfection.

#### RNA Detection

Real time PCR was used to detect RNA expression of Gab1 and VEGFR-2. Primer: Gab1-F: ATCAGAAACGCCAGCGAAGA, Gab1-R: TCAGATACCACAAAGCACCA, VEGFR-2-F: CCAATAATCAGAGTGGCAGTG, and VEGFR-2-R: ATAGACATAAATGACCGAGGC. Results were analyzed using ExicyclerTM 96 (BIONEER, South Korea). RT-PCR was used to detect RNA level of MMP-9 after interference of Gab1 and VEGFR-2. Primer: MMP-9-F: GCTACGTGACCTATGACATCCT, MMP-9-R: TCCTCCAGAACAGAATACCAGT. Each experiment was repeated three times.

#### Protein Detection

Corresponding antibodies were used to detect proteins expression of Gab1, p-Gab1, VEGFR-2, p-VEGFR-2, Akt, p-Akt and MMP-9. Each experiment was repeated three times.

### MTT Assay

Cells were digested for counting and further diluted to a cell suspension of 5.0×10^4^/ml. Cells were inoculated into a 96-well cell culture plate at 200 μl/well, (i.e., approximately 1×10^4^/well). Five wells were set for each group in duplicate. The plate was placed into a 37°C, 5% CO2 incubator for 24 h culture. A total of 5 mg/ml MTT was added (20 μl/well) and placed into a 37°C incubator for a 4 h-6 h incubation. Cells were centrifuged at 1000 rpm for 10 min. The supernatant was carefully removed with a pipette and 200 μl DMSO was added to dissolve crystals. OD of the resultant solution was determined at 490 nm using a microplate reader. The assay was repeated three times.

### Cell Cycle Detection with Propidium Iodide Staining and Flow Cytometry

Cells in the logarithmic growth phase were used for experiments. Cells were cultured, digested and collected at a fusion rate of 90% and fixed with 70% ethanol for 2 h at 4°C. Fixed cells were centrifuged at 800 rpm for 5 min and collected. The supernatant was discarded. A total of 500 μl staining buffer solution was added into each tube containing cell sample to slowly and completely re-suspend the cells. A total of 25 μl propidium iodide staining solution was first added to the resultant suspension, followed by 10 μl RNase A and mixed evenly. The mixture was incubated in the dark for 30 min at 37°C and tested by flow cytometry. The experiment was repeated three times.

### Apoptosis Detection with Annexin V and Propidium Iodide

Cells in the logarithmic growth phase were used for experiments. The cells were centrifuged at 800 rpm for 5 min and collected. The supernatant was carefully removed with a pipette, and the remaining cells were washed twice with PBS and centrifuged at 800 rpm for 5 min and collected. The supernatant was carefully removed with a pipette, with about 50 μl PBS remaining. Thereafter, 500 μl binding buffer was added into each tube to gently re-suspend the cells. The resultant suspension was added with 5 μl annexin V-FITC and 5 μl propidium iodide and mixed evenly. The mixture was incubated in the dark for 15 min at room temperature and tested by flow cytometry. The experiment was repeated three times.

### Cell Invasive Capacity Detection with Transwell

Establishment of transwell chamber model: Matrigel was preserved at 4°C overnight for thawing and diluted with serum-free medium (SFM) at a ratio of 1:3 on a super-clean bench. A total of 20 μl of the resultant dilution was added into Transwell chambers on a 24-well plate and gelated for 2 h at 37°C. Cell treatment: After the culture media was removed for each group of transfected cells and the control group, the cells were digested with 0.25% trypsin to produce a single cell suspension. The suspension was diluted to a cell suspension of 1×10^5^/ml for use after cell counting. A total of 800 μl culture medium containing 20% FBS was added into the coated lower Transwell chambers, and 200 μl of cell suspension was added into the upper Transwell chambers at 5×10^4^/well for all groups. The plate was cultured for 24 h. Fixation and staining: Transwell chambers were removed and gently washed with PBS. Cells on the upper layer of the microporous membrane were removed with a cotton swab, fixed with paraformaldehyde (PFA) for 20 min at room temperature, and stained with hematoxylin staining solution for 3 min. Cell counting: Cells migrating to the lower layer of the microporous membrane were counted in 5 fields under an inverted microscope (×200) for each sample and the results were averaged. The experiment was repeated three times.

### Statistical Analysis

All data were presented as the mean ± standard deviation (SD). The *t* test (for data with a normal distribution) or Mann-Whitney *U* test (for data with a non-normal distribution) was used for mean comparison between two groups, and Spearman rank test for correlation analysis was used to compare means between various groups. IBM SPSS19 software package was used for statistical analysis. Differences were regarded as statistically significant when *P*<0.05.

## Results

### VEGFR-2, Gab1 and MMP-9 Are Correlated with Hilar Cholangiocarcinoma

**Figure 1:**
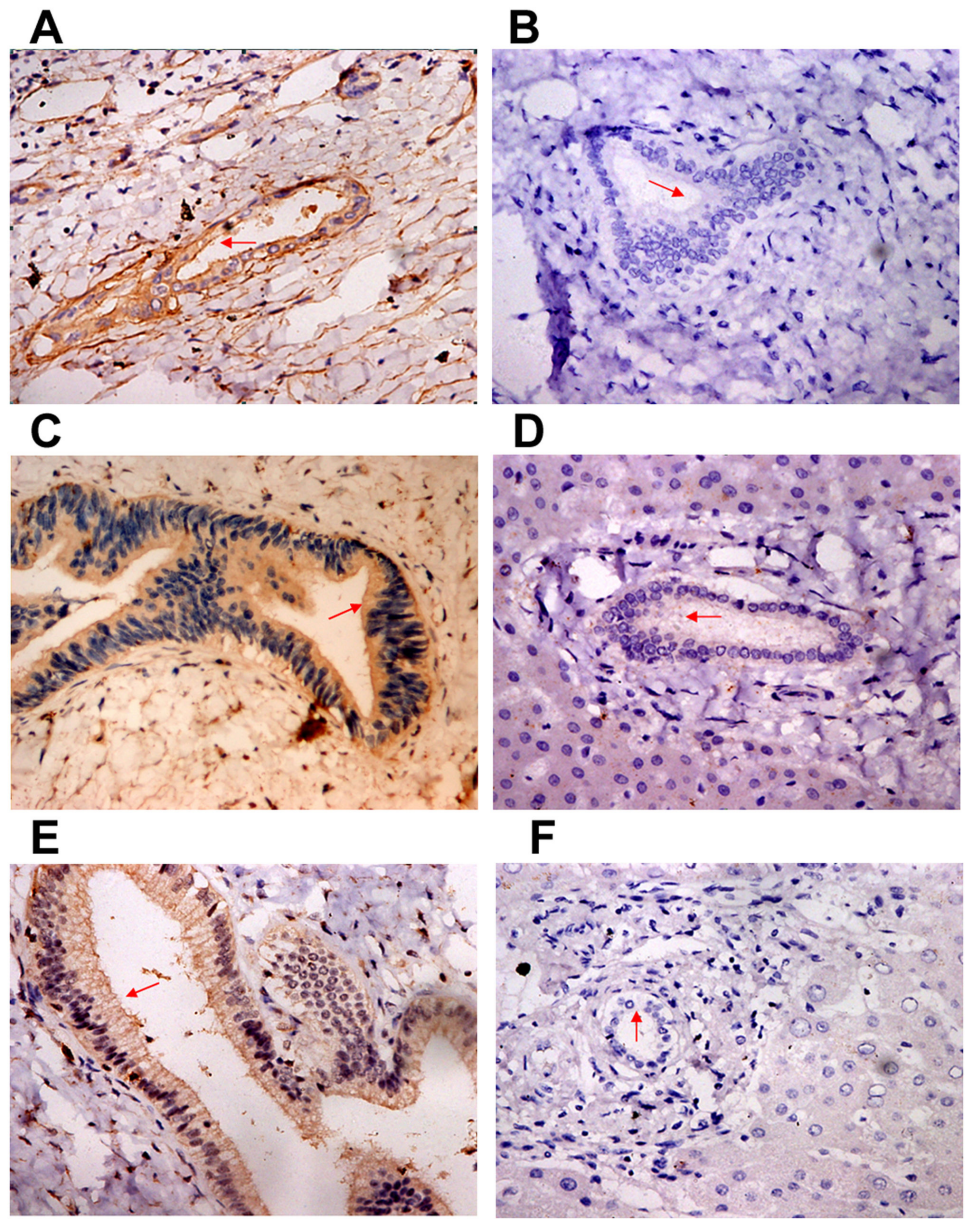
Immunohistochemistry. VEGFR-2, Gab1 and MMP-9 expression in hilar cholangiocarcinoma tissues and biliary duct tissues with chronic inflammation were detected using immunohistochemical methods. Results were expressed as mean OD. Comparison of the mean OD showed that VEGFR-2, Gab1 and MMP-9 expression levels in hilar cholangiocarcinoma tissues (A, C, E) were significantly higher than those in biliary duct tissues with chronic inflammation (B, D, F) (**P<0.01).

According to a large number of literature reports, the growth, invasion and metastasis of malignant tumors are closely related with VEGFR-2 [17-20] and MMP-9 [21,22], and the Gab1-mediated VEGFR-2 signaling pathway can promote cell growth and invasion [23]. We presumed that VEGFR-2, Gab1 and MMP-9 likely play roles in hilar cholangiocarcinoma. As shown in Figure 1 (A, C, E), VEGFR-2, Gab1 and MMP-9 were all highly expressed in hilar cholangiocarcinoma tissues compared to biliary duct tissues with chronic inflammation. Table 1 shows OD and statistical analysis for all pathological sections. Mean VEGFR-2, Gab1 and MMP-9 ODs in hilar cholangiocarcinoma tissues were 0.2174±0.009, 0.2193±0.0092 and 0.2207±0.0106, respectively, which were significantly higher than the ODs in biliary duct tissues with chronic inflammation (0.175±0.0024, 0.1761±0.0046 and 0.1748±0.0026, respectively). As shown in Table 2, VEGFR-2 expression in hilar cholangiocarcinoma tissues was positively correlated with lymphatic metastasis in patients, but was not associated with the sex, age, differentiation, tumor size, invasion depth and TNM stage of patients. Gab1 and MMP-9 expression in hilar cholangiocarcinoma tissues were negatively correlated with differentiation and positively correlated with lymphatic metastasis in patients, but not associated with the sex, age, tumor size, invasion depth and TNM stage of patients. As shown in Table 3, VEGFR-2, Gab1 and MMP-9 expression levels in hilar cholangiocarcinoma tissues were positively correlated with each other. These results demonstrate that VEGFR-2, Gab1 and MMP-9 are all highly expressed in hilar cholangiocarcinoma tissues and closely related to the malignant biological behaviors of hilar cholangiocarcinoma and are correlated with each other.

**Table 1:** OD analysis of VEGFR-2, Gab1 and MMP-9 expression in hilar cholangiocarcinoma tissues and biliary duct tissues with chronic inflammation.

Item	Hilar cholangiocarcinoma mean OD	Biliary duct with chronic inflammation mean OD	*P*
VEGFR-2	0.2174 ± 0.009	0.175 ± 0.0024	0.000**
Gab1	0.2193 ± 0.0092	0.1761 ± 0.0046	0.000**
MMP-9	0.2207 ± 0.0106	0.1748 ± 0.0026	0.000**

** *P*<0.01

**Table 2:** Relationship between VEGFR-2, Gab1 and MMP-9 expression in hilar cholangiocarcinoma tissues and patient clinical and pathological parameters.

Item	VEGFR-2 mean OD	*P*	Gab1 mean OD	*P*	MMP-9 mean OD	*P*
Sex						
Male	0.2171 ± 0.0092	0.697	0.2192 ± 0.0078	0.249	0.22 ± 0.0108	0.498
Female	0.2181 ± 0.0085		0.2219 ± 0.0115		0.2221 ± 0.0103	
Age (years)						
≤60	0.2165 ± 0.0095	0.506	0.2191 ± 0.0102	0.838	0.22 ± 0.0113	0.077
>60	0.2182 ± 0.0085		0.2196 ± 0.0083		0.2231 ± 0.0104	
Differentiation						
High	0.2155 ± 0.0097	0.053	0.217 ± 0.0084	0.021*	0.218 ± 0.0115	0.023*
Moderate-low	0.2203 ± 0.0069		0.2229 ± 0.0093		0.2247 ± 0.0078	
Tumor size						
<2cm	0.2164 ± 0.0075	0.522	0.2203 ± 0.0088	0.814	0.2217 ± 0.0087	0.839
≥2cm	0.2181 ± 0.0099		0.2196 ± 0.0102		0.2209 ± 0.0119	
Invasion depth^1^						
T1+T2	0.218 ± 0.009	0.647	0.2201 ± 0.0087	0.557	0.223 ± 0.0092	0.127
T3	0.2169 ± 0.0091		0.2186 ± 0.0096		0.2185 ± 0.0116	
Lymphatic metastasis						
No	0.2153 ± 0.009	0.024*	0.2172 ± 0.0081	0.026*	0.2174 ± 0.0095	0.003**
Yes	0.2209 ± 0.0079		0.2235 ± 0.0097		0.226 ± 0.0104	
TNM stage^1^						
Ⅰ+Ⅱ	0.2172 ± 0.0087	0.909	0.2185 ± 0.0078	0.539	0.2212 ± 0.0089	0.771
Ⅲ+Ⅳ	0.2175 ± 0.0093		0.2199 ± 0.0101		0.2203 ± .0118	

^1^ Adapted from International Union Against Cancer (UICC): TNM Classification of malignant tumours. Sixth edition. 2002; 81–89.

*P<0.05, **P<0.01

**Table 3:** Correlation between Gab1 and VEGFR-2, Gab1 and MMP-9, and VEGFR-2 and MMP-9 expression in hilar cholangiocarcinoma tissues.

Item	Hilar cholangiocarcinoma mean OD	Gab1 and VEGFR-2^1^	Gab1 and MMP-9^1^	VEGFR-2 and MMP-9^1^
VEGFR-2	0.2174 ± 0.009	*r*_*s*_=0.623 *P*=0.000**	*r*_*s*_=0.477 *P*=0.000**	*r*_*s*_=0.592 *P*=0.000**
Gab1	0.2193 ± 0.0092			
MMP-9	0.2207 ± 0.0106			

^1^ Spearman rank test was used for correlation analysis.

** *P*<0.01

### Gab1 and VEGFR-2 Have an Effect via the PI3K/Akt Pathway and Regulate MMP-9 Expression in ICBD-1 Cells

Evaluation of hilar cholangiocarcinoma specimens showed that VEGFR-2, Gab1 and MMP-9 were all closely correlated with hilar cholangiocarcinoma and its malignant biological behaviors and were related to each other. Because the VEGFR-2/Gab1/PI3K/Akt signaling pathway promotes cell growth and invasion [23] and MMP-9 is one important downstream target protein of PI3K/Akt [24], we presumed that they may play roles in hilar cholangiocarcinoma. To validate this assumption, we designed 4 Gab1 or VEGFR-2 interference sequences and 1 irrelevant interference sequence (NC) using software, detected interference efficiency by real time PCR and Western Blot (data not shown) and selected the Gab1 siRNA3 and VEGFR-2 siRNA3 with the highest interference efficiency for transfection into ICBD-1 cells.

**Figure 2:**
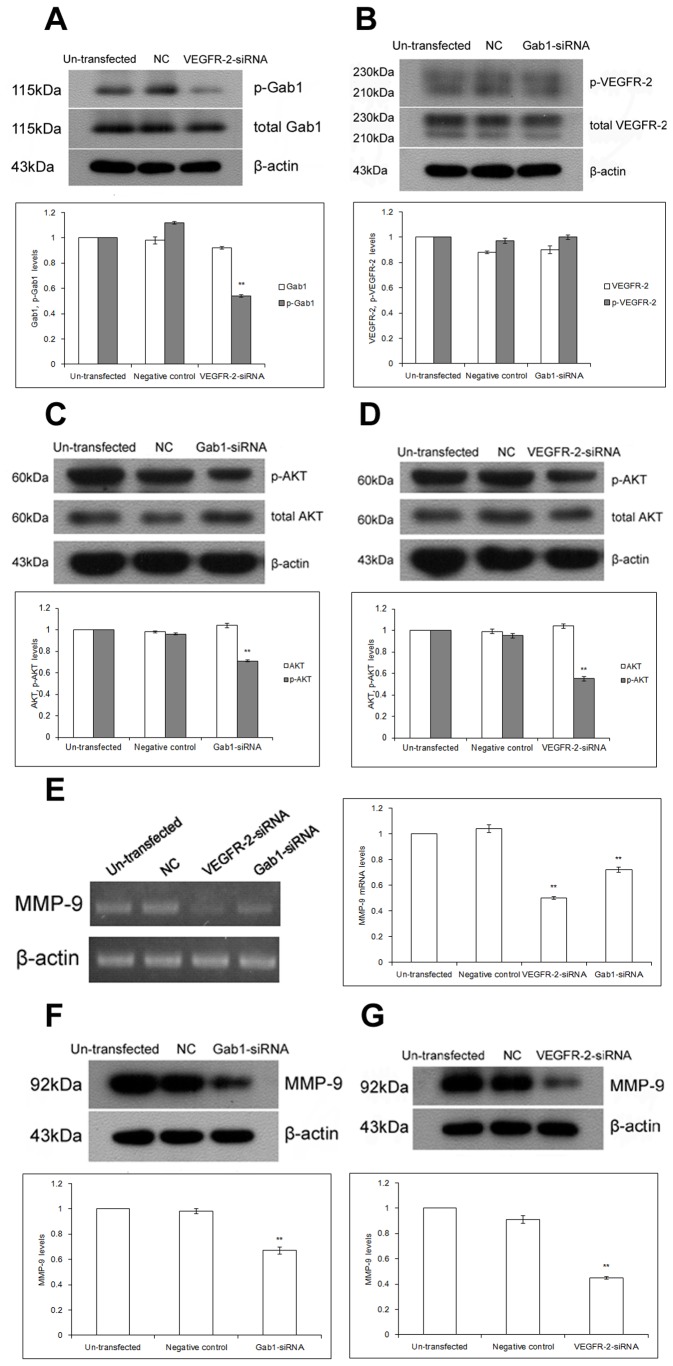
Changes in PI3K/Akt pathway activity and MMP-9 expression after RNA interference. A: Compared with the NC group (controls transfected by 1 irrelevant interference sequence), significantly decreased expression of p-Gab1, but no obvious change in total Gab1 expression in ICDB-1 cells were observed after VEGFR-2 siRNA interference, **P<0.01. Compared with the un-transfected group, there were no significant changes in Gab1 and p-Gab1 expression in ICBD-1 cells within the NC group. B: Compared with the NC group, there were no significant changes in VEGFR-2 and p-VEGFR-2 expression in ICBD-1 cells after Gab1 siRNA interference. C: Compared with the NC group, significantly decreased expression of p-Akt, but no obvious change in total Akt expression in ICDB-1 cells were observed after Gab1 siRNA interference, **P<0.01. Compared with the un-transfected group, there were no significant changes in Akt and p-Akt expression in ICBD-1 cells within the NC group. D: Compared with the NC group, significantly decreased p-Akt expression in ICDB-1 cells was observed after VEGFR-2 siRNA interference, **P<0.01. E: Compared with the NC group, significantly decreased MMP-9 mRNA level in ICDB-1 cells was observed after Gab1 siRNA or VEGFR-2 siRNA interference, **P<0.01. Compared with the un-transfected group, there was no significant change in MMP-9 level in ICBD-1 cells within the NC group. F: Compared with the NC group, significantly decreased expression of MMP-9 in ICDB-1 cells was observed after Gab1 siRNA interference, **P<0.01. G: Compared with the NC group, significantly decreased expression of MMP-9 in ICDB-1 cells was observed after VEGFR-2 siRNA interference, **P<0.01.

VEGFR-2 was reported an upstream protein of Gab1 in endothelial cells [23,25,26] and our data showed that VEGFR-2 and Gab1 were positively correlated in hilar cholangiocarcinoma, so we detected whether VEGFR-2 was the upstream protein of Gab1 in hilar cholangiocarcinoma cells. As shown in Figure 2A, there was no significant change in total Gab1 expression, but p-Gab1 expression was reduced in ICBD-1 cells after VEGFR-2 siRNA interference compared with the NC group. However, there were no significant changes in both total VEGFR-2 expression and p-VEGFR-2 expression in ICBD-1 cells after Gab1 siRNA interference compared with the NC group (Figure 2B). These results indicate that VEGFR-2 is the upstream protein of Gab1 in ICBD-1 cells and regulates the phosphorylation level of Gab1.

Changes in PI3K/Akt pathway activity in hilar cholangiocarcinoma tumor cells after down-regulation of Gab1 and VEGFR-2 were also determined. As shown in Figure 2C, there was no significant change in total Akt expression, but p-Akt expression was reduced in ICBD-1 cells after Gab1 siRNA interference compared with the NC group, suggesting reduced PI3K/Akt pathway activity. Compared with the un-transfected group, no significant changes were observed in both total Akt expression and p-Akt expression in the NC group. Similarly, as shown in Figure 2D, PI3K/Akt pathway activity was decreased in ICBD-1 cells after VEGFR-2 siRNA interference. These results show that either Gab1 or VEGFR-2 interference down-regulates PI3K/Akt pathway activity in ICBD-1 cells.

Finally, we detected MMP-9 levels in hilar cholangiocarcinoma tumor cells after down-regulation of Gab1 and VEGFR-2. As shown in Figure 2E, F and G, mRNA and protein levels of MMP-9 were both reduced after Gab1 siRNA or VEGFR-2 siRNA interference compared with the NC group in ICBD-1 cells, indicating that either Gab1 or VEGFR-2 interference down-regulated MMP-9 expression in ICBD-1 cells. All these results demonstrate a potential role of the VEGFR-2/Gab1/PI3K/Akt pathway in ICBD-1 cells.

### Decreased Growth Capability of ICBD-1 Cells after Gab1 and VEGFR-2 Down-Regulation by Interference

**Figure 3:**
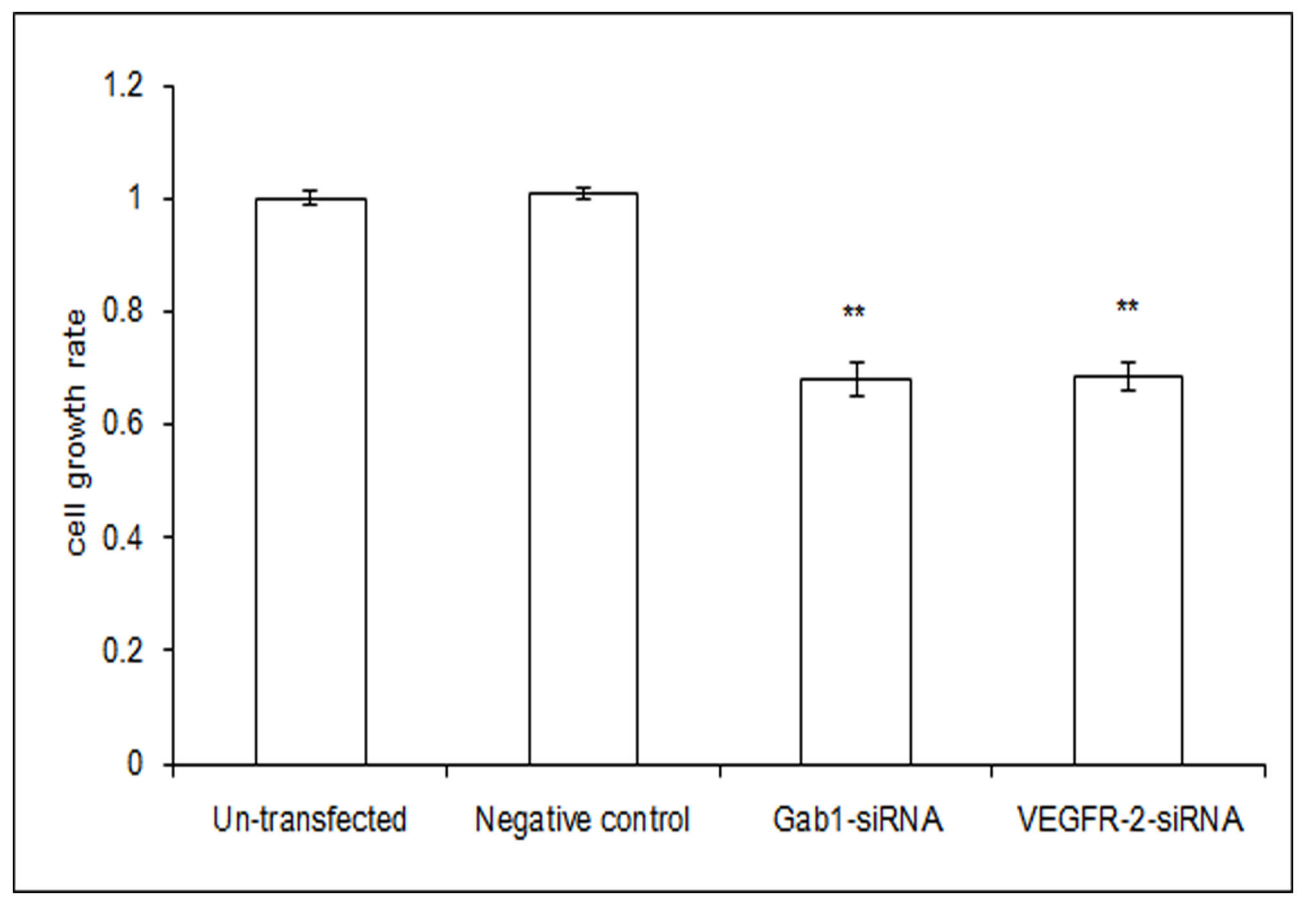
Cell growth rate. Cell growth in each group was detected using the MTT assay. Compared with the NC group (controls transfected by 1 irrelevant interference sequence), the growth rate of ICBD-1 cells was significantly decreased after Gab1 siRNA interference or VEGFR-2 siRNA interference, **P<0.01. Compared with the un-transfected group, there was no significant change in the growth rate of ICBD-1 cells within the NC group.

The VEGFR-2/Gab1/PI3K/Akt pathway plays a role in ICBD-1 cells, which has not been previously reported. We further investigated cell growth using interference fragments. We selected Gab1 siRNA and VEGFR-2 siRNA with the highest interference efficiency to transfect into ICBD-1 cells and used the MTT colorimetry assay to determine cell growth. As shown in Figure 3, growth capacity of ICBD-1 cells was significantly decreased after Gab1 siRNA or VEGFR-2 siRNA interference compared with the NC group. Compared with the un-transfected group, no significant changes in cell growth were observed in the NC group. These findings demonstrate that the growth capacity of ICBD-1 cells is weakened after Gab1 or VEGFR-2 down-regulation by interference with decreased PI3K/Akt pathway activity, indicating that the VEGFR-2/Gab1/PI3K/Akt pathway up-regulates the growth capacity of ICBD-1 cells and that interference can result in decreased cell growth capacity.

### Arrested in G1 Phase of ICBD-1 Cell Growth after Gab1 and VEGFR-2 Down-Regulation by Interference

**Figure 4:**
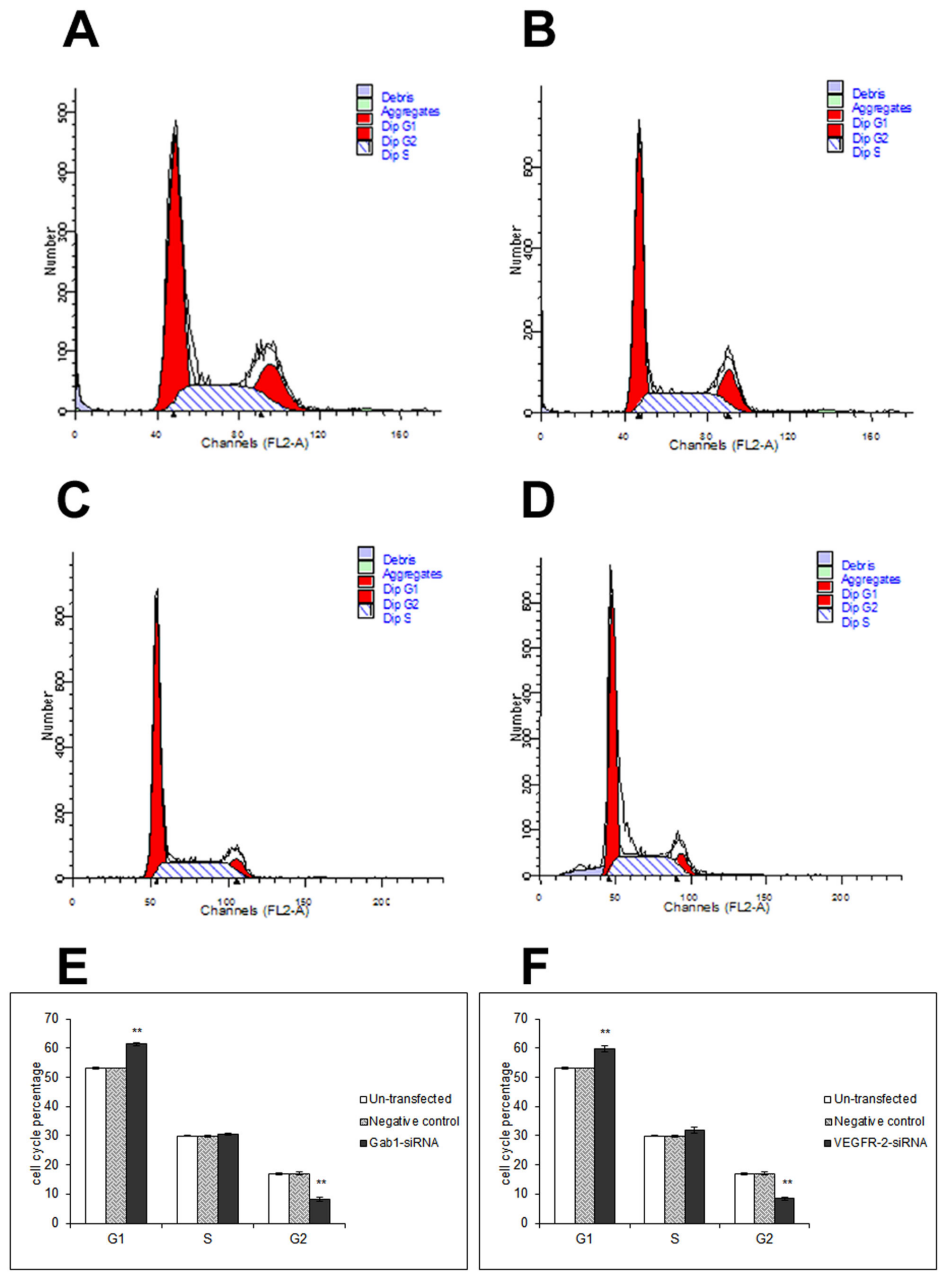
Cell cycle changes after RNA interference. Cell cycle changes in each group were detected using an RNA interference technique, PI staining and flow cytometry. A-D: Flow cytometric diagrams; A: un-transfected group, B: NC group (controls transfected by 1 irrelevant interference sequence), C: Gab1 siRNA group, D: VEGFR-2 siRNA group. Compared with the NC group, the proportion of ICBD-1 cells in G1 phase was increased, **P<0.01, G2 phase was decreased, **P<0.01, and S phase demonstrated no significant change after Gab1 siRNA or VEGFR-2 siRNA interference. Compared with the un-transfected group, no significant changes in the cell cycle were observed in the NC group. Figures E and F show the statistical results of three experiments, respectively. These results indicate that Gab1 and VEGFR-2 can affect cell cycle changes in ICBD-1 cells and their down-regulation could arrest ICBD-1 cell growth in the G1 phase and weaken cell growth, which is consistent with MTT test results.

We further investigated the mechanism of VEGFR-2/Gab1/PI3K/Akt regulation of ICBD-1 cell growth. We selected Gab1 siRNA and VEGFR-2 siRNA with the highest interference efficiency to transfect into ICBD-1 cells. Figure 4A, B, C and D show that compared with the NC group an increased proportion of ICBD-1 cells in the G1 phase, a decreased proportion of ICBD-1 cells in the G2 phase, and no significant change of ICBD-1 cells in the S phase after Gab1 siRNA or VEGFR-2 siRNA interference. Compared with the un-transfected group, no significant changes in the cell cycle were observed in the NC group. These findings demonstrate changes in the cell cycle of ICBD-1 cells after Gab1 or VEGFR-2 down-regulation by interference (i.e., the growth of all cells are arrested in G1 phase, thus weakening cell growth capacity, which is consistent with MTT test results).

### Increased Apoptosis Capacity of ICBD-1 Cells after Gab1 and VEGFR-2 Down-Regulation by Interference

**Figure 5:**
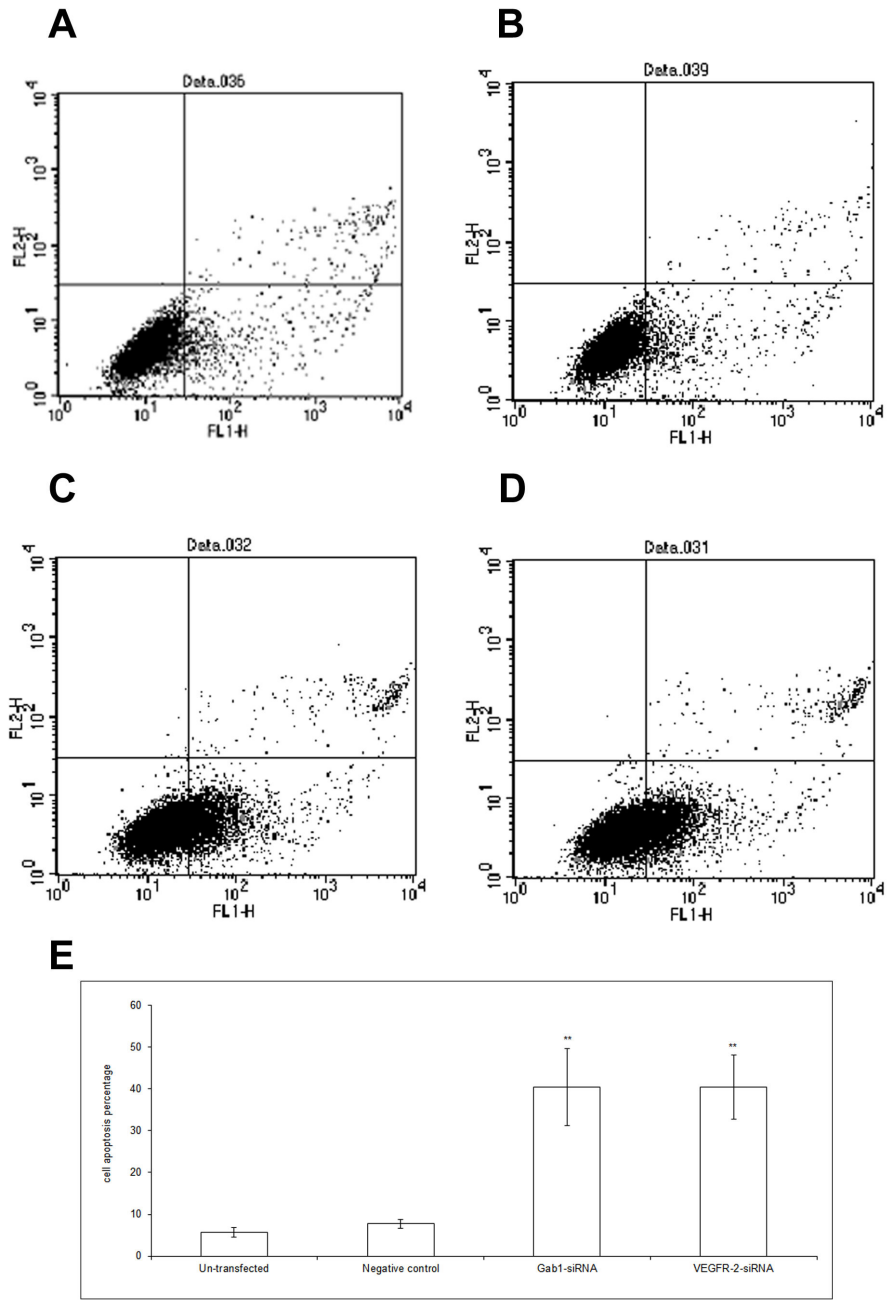
Changes in cell apoptosis capacity after RNA interference. Cell apoptosis in each group were detected using an RNA interference technique and AV/PI staining. A-D: Flow cytometric diagrams; A: un-transfected group, B: NC group (controls transfected by 1 irrelevant interference sequence), C: Gab1 siRNA group, D: VEGFR-2 siRNA group. E: Statistical results of three experiments. The proportion of apoptotic ICBD-1 cells increased in the early stage after Gab1 siRNA or VEGFR-2 siRNA interference compared with NC group, **P<0.01. Compared with the un-transfected group, there were no significant changes in early cell apoptosis in the NC group.

In addition to regulating ICBD-1 cell growth, the VEGFR-2/Gab1/PI3K/Akt pathway may participate in regulating cell apoptosis. We selected Gab1 siRNA and VEGFR-2 siRNA with the highest interference efficiency to transfect into ICBD-1 cells. Figure 5A, B, C and D show that the proportion of apoptotic ICBD-1 cells was significantly increased after Gab1 siRNA or VEGFR-2 siRNA interference compared with NC group. Compared with the un-transfected group, there were no significant changes in cell apoptosis in the NC group. The above results demonstrate that the number of apoptotic ICBD-1 cells increases after Gab1 and VEGFR-2 down-regulation by interference, indicating that the VEGFR-2/Gab1/PI3K/Akt pathway can inhibit apoptosis of ICBD-1 cells and that its suppression can result in increased apoptosis.

### Decreased Invasive Capacity of ICBD-1 Cells after Gab1 and VEGFR-2 Down-Regulation by Interference

**Figure 6:**
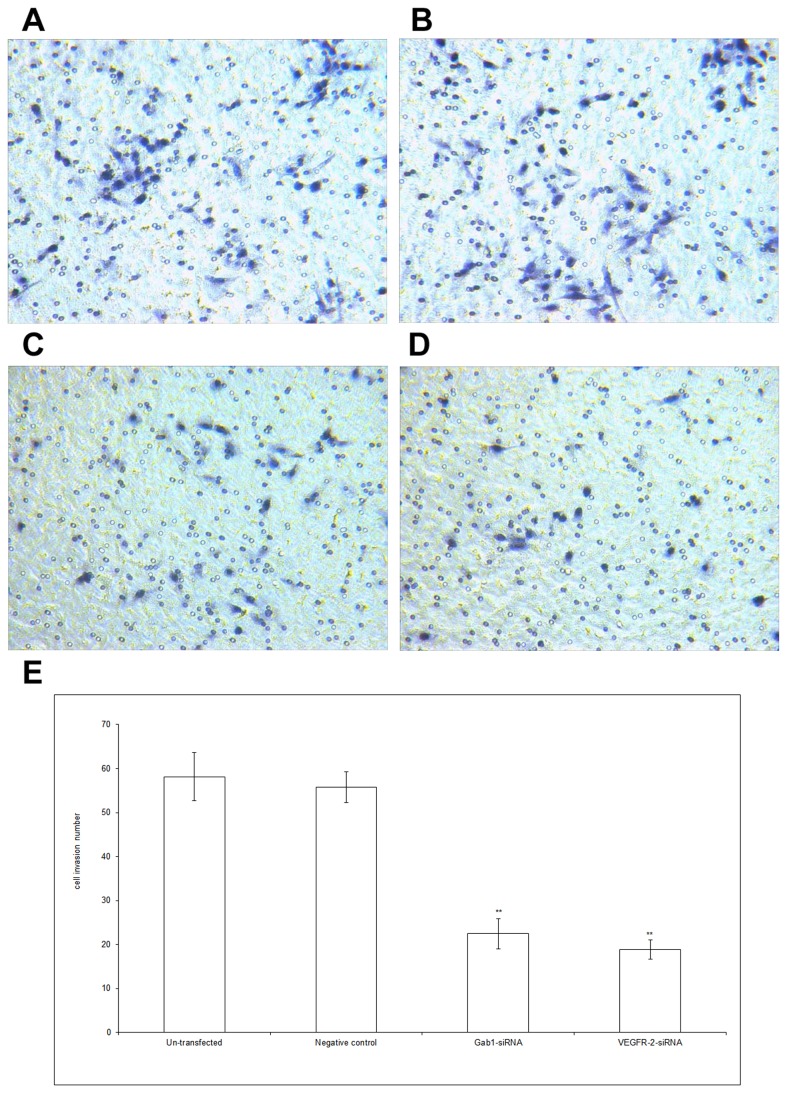
Changes in cell invasive capacity after RNA interference. Changes in cell invasive capacity in each group were detected using a RNA interference technique and Transwell method. A-D: Cell invasion images; A: un-transfected group, B: NC group (controls transfected by 1 irrelevant interference sequence), C: Gab1 siRNA group, D: VEGFR-2 siRNA group. E: Statistical results of three experiments. Compared with NC group, the invasive capacity of ICBD-1 cells decreased after Gab1 siRNA or VEGFR-2 siRNA interference, **P<0.01. Compared with the un-transfected group, no significant changes in cell invasive capacity were observed in the NC group.

Invasive capacity is essential for the malignant biological behaviors of tumors. Experimental results suggest that the VEGFR-2/Gab1/PI3K/Akt pathway can regulate the malignant biological behaviors (i.e., growth and apoptosis) of ICBD-1 cells. To investigate whether this pathway participates in regulating tumor cell invasion, we selected Gab1 siRNA and VEGFR-2 siRNA with the highest interference efficiency to transfect into ICBD-1 cells. As shown in Figure 6A, B, C and D, the invasive capacity of ICBD-1 cells decreased after Gab1 siRNA or VEGFR-2 siRNA interference compared with the NC group. Compared with the un-transfected group, no significant changes in cell invasive capacity were observed in the NC group. The invasive capacity of ICBD-1 cells and MMP-9 levels decreased after Gab1 and VEGFR-2 down-regulation by interference, suggesting that the VEGFR-2/Gab1/PI3K/Akt pathway could up-regulate the invasive capacity of ICBD-1 cells and that interference could lower the invasive capacity of the cells.

In summary, experimental results demonstrate that VEGFR-2, Gab1 and MMP-9 are closely correlated with hilar cholangiocarcinoma and that Gab1 or VEGFR-2 up-regulates growth and invasion and down-regulates apoptosis in ICBD-1 cells via the PI3K/Akt pathway. This the first study to describe correlations between Gab1 and hilar cholangiocarcinoma and to provide preliminary exploration of the biological role of the VEGFR-2/Gab1/PI3K/Akt pathway.

## Discussion

Hilar cholangiocarcinoma, the most common malignancy of the biliary system [2], is characterized by insidious onset. It has a low radical surgical resection rate because early diagnosis is difficult and most clinical patients have middle or advanced stage with a high risk of infiltration and metastasis by the time they seek medical intervention [5,6]. Hilar cholangiocarcinoma easily relapses after surgery and is insensitive to radiotherapy and chemotherapy [4,7-9], with a low overall 5-year survival rate. At present, an effective comprehensive therapy for hilar cholangiocarcinoma is lacking, and infiltration and metastasis mechanisms have not been well studied. As a result, clinical treatment technologies have shown minimal improvement. All of the above factors render hilar cholangiocarcinoma a major challenge in general surgery.

As a multi-substrate adapter protein, Gab1 has a highly conservative PH structural domain near the N-terminal, and its middle 2/3 section is a proline-rich structural domain with multiple SH2 and SH3 binding sites and Met coupling sequences [12]. This PH structural domain is capable of binding PIP3 on the cell membrane and anchors Gab1 at the internal surface of the cell membrane, further increasing the chance and time for interactions between Gab1 and receptors. SH2 binding sites can bind with signal molecules containing YXXP sequences, such as PI3K subunit p85, SHP2, PLC-γ and Crk, to exert a biological effect [12]. Gab1 has been reported to be closely involved in the occurrence, invasion and metastasis of tumors, particularly gastrointestinal tumors [15,16,27,28]. Seiden-Long et al. [27] reported that Gab1 over-expression could stimulate colon carcinoma cell growth with high expression of Met. Gab1 is also required for cellular differentiation in Caco-2 colon carcinoma [28]. In this study, we reviewed 49 hilar cholangiocarcinoma cases. Using immunohistochemical analysis, we detected high expression of Gab1 in hilar cholangiocarcinoma tissues and further confirmed that Gab1 was correlated with differentiation and lymphatic metastasis in patients by combining other clinical information (Figure 1C, Table 1 and 2). As a major metastatic pathway of hilar cholangiocarcinoma, lymphatic metastasis and differentiation are two important factors for poor prognosis [4,29-31]. Thus, we suspected a close relationship between high Gab1 expression and malignant biological behaviors (e.g., invasion and metastasis) of hilar cholangiocarcinoma.

Based on common knowledge, VEGFR-2 can mediate neogenesis of tumor blood vessels and the lymphatic system, distal lymphatic metastasis of tumor cells [17,18] and promote the occurrence, invasion and metastasis of tumors [19,20,32]. Interrupting binding between VEGF and VEGFR-2 can significantly inhibit the growth and metastasis of tumors [33]. MMP-9 is capable of degrading collagen IV, a main component of the ECM and basement membrane, and thus plays an important role in the invasion and metastasis of tumor cells [21,22,34,35]. Onodera et al. [36] reported that MMP-9 was highly expressed in cholangiocarcinoma and promoted tumor invasion and metastasis. Our results showed that VEGFR-2 and MMP-9 were both highly expressed and VEGFR-2 was correlated with lymphatic metastasis, while MMP-9 was correlated with differentiation and lymphatic metastasis (Figure 1A and 1E, Table 1 and 2). Further analysis demonstrated that VEGFR-2, Gab1 and MMP-9 were positively correlated with each other in solid hilar cholangiocarcinoma tumors (Table 3), indicating potential interactions between VEGFR-2, Gab1 and MMP-9. These interactions might be closely related with the growth and invasion of hilar cholangiocarcinoma.

Growth, invasion and metastasis of tumors are closely correlated with the PI3K/Akt signaling pathway. This pathway can regulate the biological behaviors of tumors in vivo via multiple mechanisms [37,38]. For example, it has a close relationship with cell cycle, apoptosis and invasion [39,40], formation of blood vessels and lymphatic vessels [41,42], and tumor prognosis [43-45]. Leelawat et al. [46] reported that the PI3K/Akt signaling pathway could advance cell invasion of cholangiocarcinoma. Highly expressed Akt has been shown to reduce the radiotherapeutic sensitivity of cholangiocarcinoma [47] and is an important factor influencing prognosis [48]. Activated Gab1 has been reported to participate in the PI3K/Akt signaling pathway [49] and can enhance PI3K/Akt activation [50]. Moreover, VEGFR-2 can increase MMP-9 expression and promote cell metastasis [51]. Gab1, an adapter protein in endothelial cells, can mediate VEGFR-2 to activate PI3K/Akt and contribute to cell invasion [23], while MMP-9 is an important downstream target protein of PI3K/Akt [24]. Because our results showed a positive relationship among VEGFR-2, Gab1 and MMP-9 in hilar cholangiocarcinoma, we proposed that the VEGFR-2/Gab1/PI3K/Akt signaling pathway was likely present in hilar cholangiocarcinoma and could influence MMP-9 expression. After Gab1 interference using the RNA interference technique, we observed a decreased expression of p-Akt in ICBD-1 cells (Figure 2C), indicating decreased activity of the PI3K/Akt signaling pathway. Further investigation of PI3K/Akt pathway participation in regulating cell growth, apoptosis and invasion revealed that cancer cell growth was suppressed (Figure 3) due to cell growth arrest in G1 phase (Figure 4C and E) (i.e., DNA duplication was inhibited); increased apoptosis capacity (Figure 5C and E) and decreased invasive capacity (Figure 6C and E) were observed. We believe that Gab1 likely regulate the malignant biological behaviors (e.g., growth, apoptosis and invasion) of hilar cholangiocarcinoma via the PI3K/Akt signaling pathway. Because VEGFR-2 is an upstream protein of Gab1 in endothelial cells [23,25,26] and VEGFR-2 and Gab1 were positively correlated in hilar cholangiocarcinoma, we examined the relationship between them in ICBD-1 cells and demonstrated that VEGFR-2 could regulate the activity of Gab1 in ICBD-1 cells (Figure 2A and B). Then, we subjected Gab1 and VEGFR-2 to RNA interference and detected decreased PI3K/Akt signaling pathway activity (Figure 2D), and down-regulated MMP-9 expression levels (Figure 2E, F, G), weakened cell growth (Figure 3 and Figure 4D, F), increased apoptosis capacity (Figure 5D and E), and decreased invasive capacity (Figure 6D and E).

By analyzing 49 hilar cholangiocarcinoma cases, we find that the adapter protein Gab1 is closely related with the occurrence, invasion and metastasis of hilar cholangiocarcinoma. A preliminary study of the mechanisms involved indicates that the VEGFR-2/Gab1/PI3K/Akt signaling pathway participates in regulating the malignant biological behaviors (e.g., growth, apoptosis and invasion) of hilar cholangiocarcinoma via the PI3K/Akt signaling pathway. This study is important for exploring the occurrence, infiltration and metastatic mechanisms of hilar cholangiocarcinoma and provides new ideas on which to base more effective therapies to improve the clinical efficacy of treating hilar cholangiocarcinoma.
